# Supplementary material for: Structural insights into photosystem II supercomplex and trimeric FCP antennae of a centric diatom Cyclotella meneghiniana
Source: Nat Commun. 2023 Dec 9;14:8164. doi: 10.1038/s41467-023-44055-8 (PMC10710467; doi:10.1038/s41467-023-44055-8)
Supplement: Supplementary file 1 — Supplementary information [file 41467_2023_44055_MOESM1_ESM.pdf]

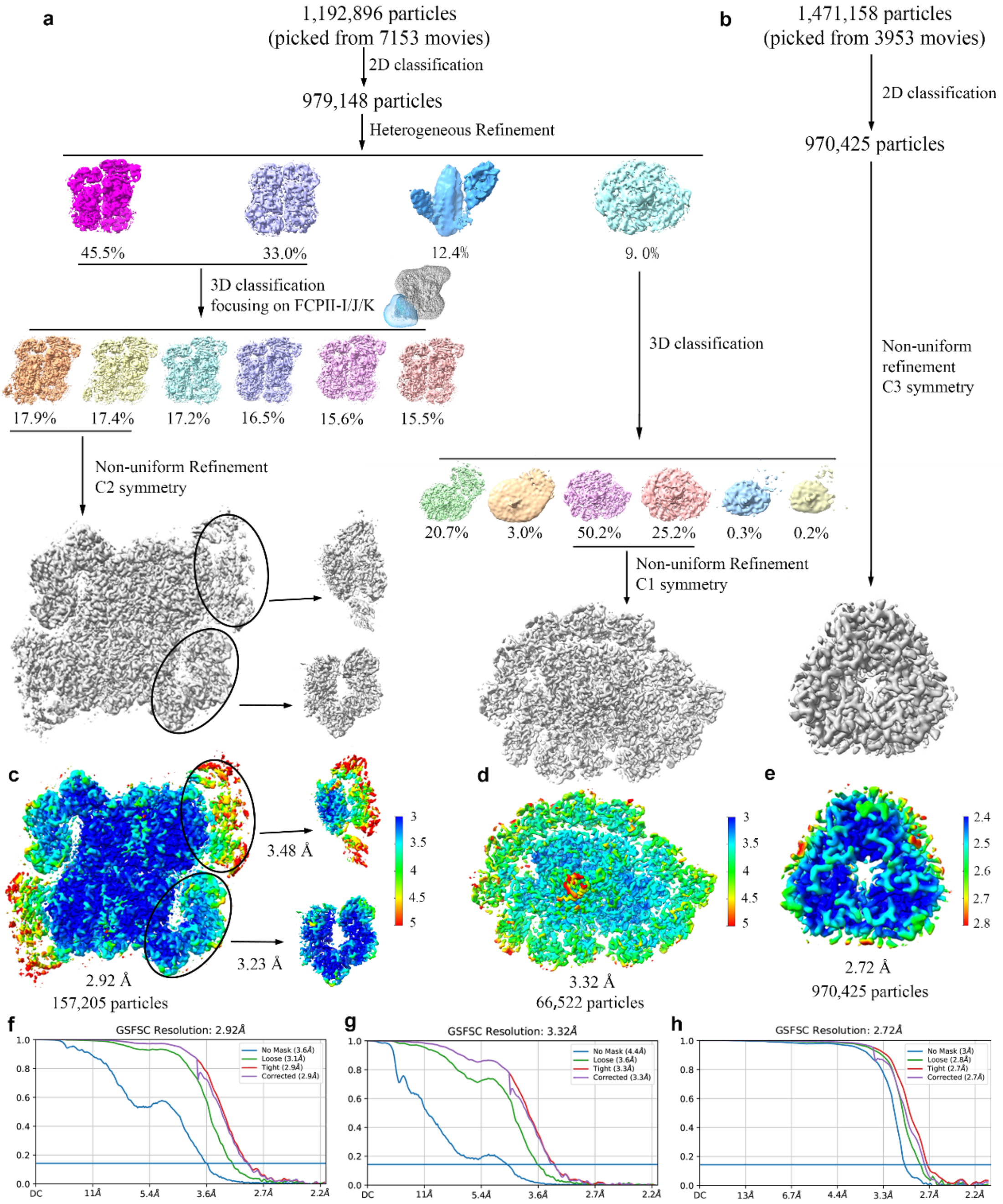

Supplementary Fig. 1. Single particle analysis of Cm-PSII-FCPII dimer, Cm-PSI-FCPI monomer and Cm-FCP trimer. a, **b**, Flowchart for the single particle analysis of Cm-PSII-FCPII dimer, Cm-PSI-FCPI monomer and Cm-FCP trimer. **c-e**, Full local resolution maps of the Cm-PSII-FCPII dimer, Cm-PSI-FCPI monomer and Cm-FCP trimer, respectively. **f-h**, The gold standard Fourier shell correlation (FSC) curve of the final density maps of Cm-PSII-FCPII dimer **(f)**, Cm-PSI-FCPI monomer **(g)** and Cm-FCP trimer **(h)**, respectively.

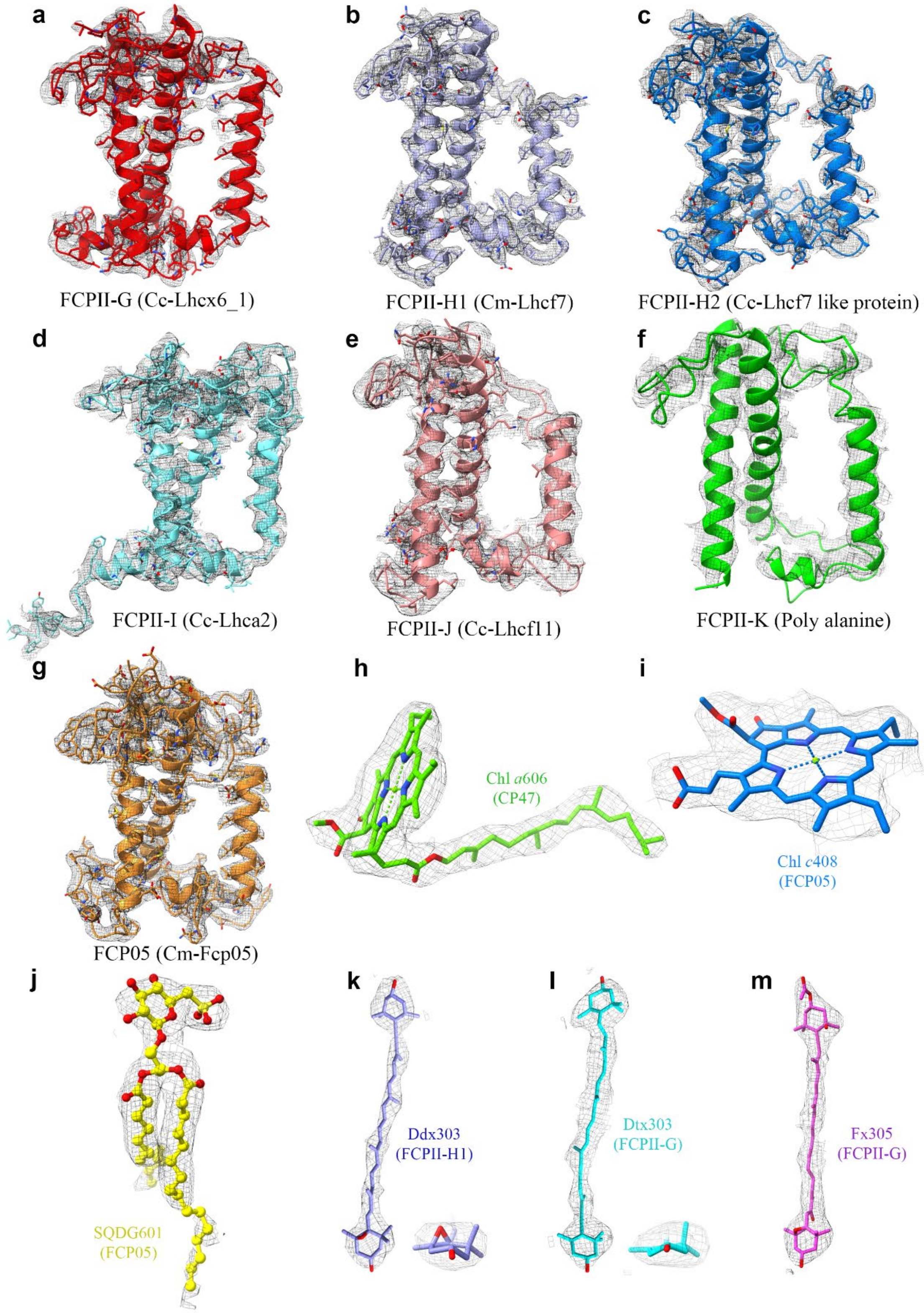

Supplementary Fig. 2. Density maps of selected subunits and cofactors.

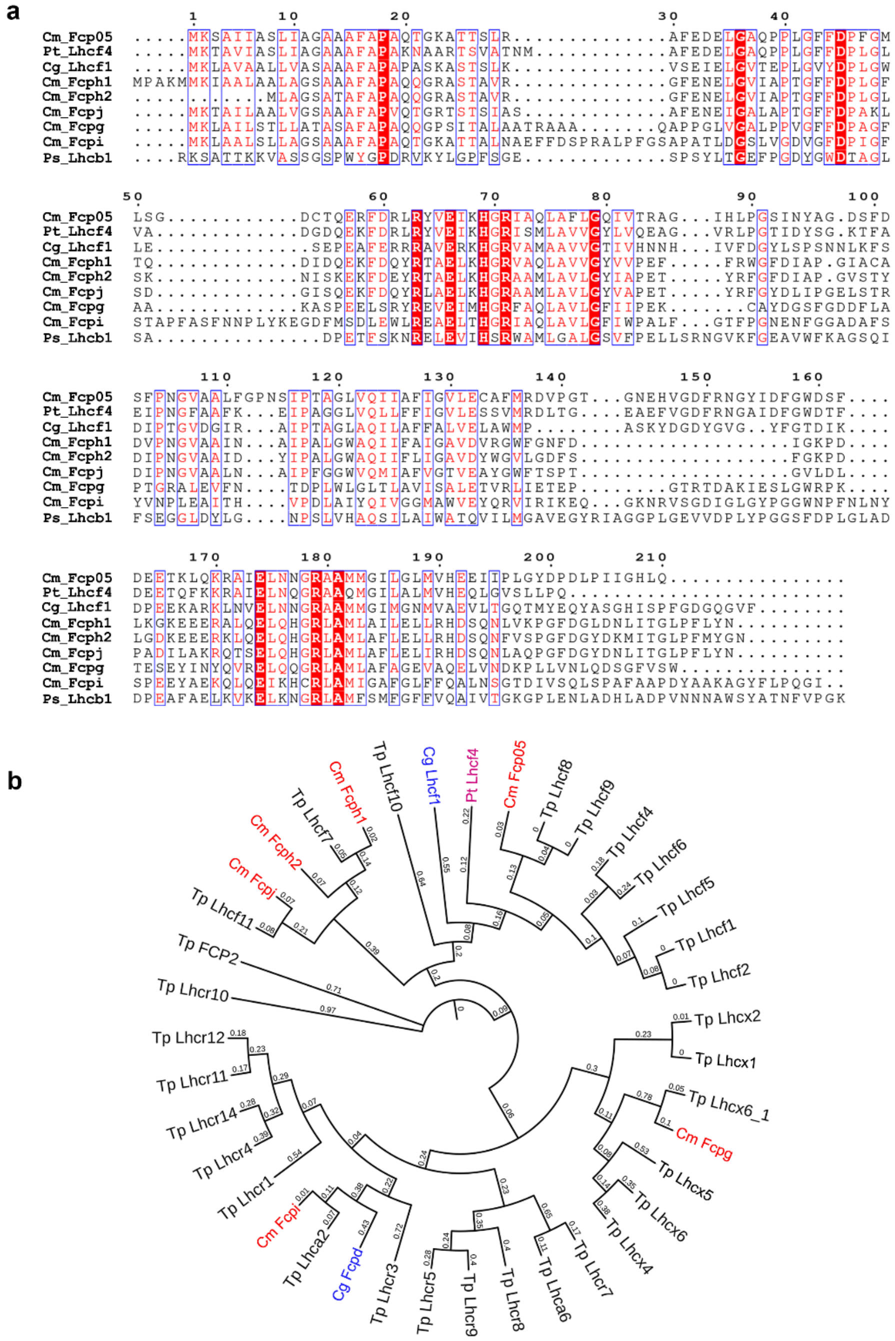

Supplementary Fig. 3. Comparison of sequences and the phylogenetic tree. a, Alignment of Cm-Fcp05 constituting the FCP trimer (Fcp05 of *C. meneghiniana*), Pt-Lhcf4, Cg-Lhcf1, Cm-Fcpg (Lhcx6_1 of *C. cryptica*), Cm-Fcph1 (Lhcf7 of *C. meneghiniana*), Cm-Fcph2 (Lhcf7 like protein of *C. cryptica*), Cm-Fcpi (Lhca2 of *C. cryptica*), Cm-Fcpj (Lhcf11 of *C. cryptica*), Ps-Lhcb1 (*P. sativum*). Sequences of Pt_Lhcf4, Cg_Lhcf1 and Ps_Lhcb1 were obtained from PDB 6A2W, 7VD6 and 2BHW, respectively. **b**, Phylogenic analysis of representative antenna subunits selected from different diatom species. The sequences of *T. pseudonana* and *P. tricornutum* were obtained from the Uniprot database. The sequences of Cg-Lhcf1 and Cg-Fcpd are extracted from two published structures, 7VD6 and 6JLU.

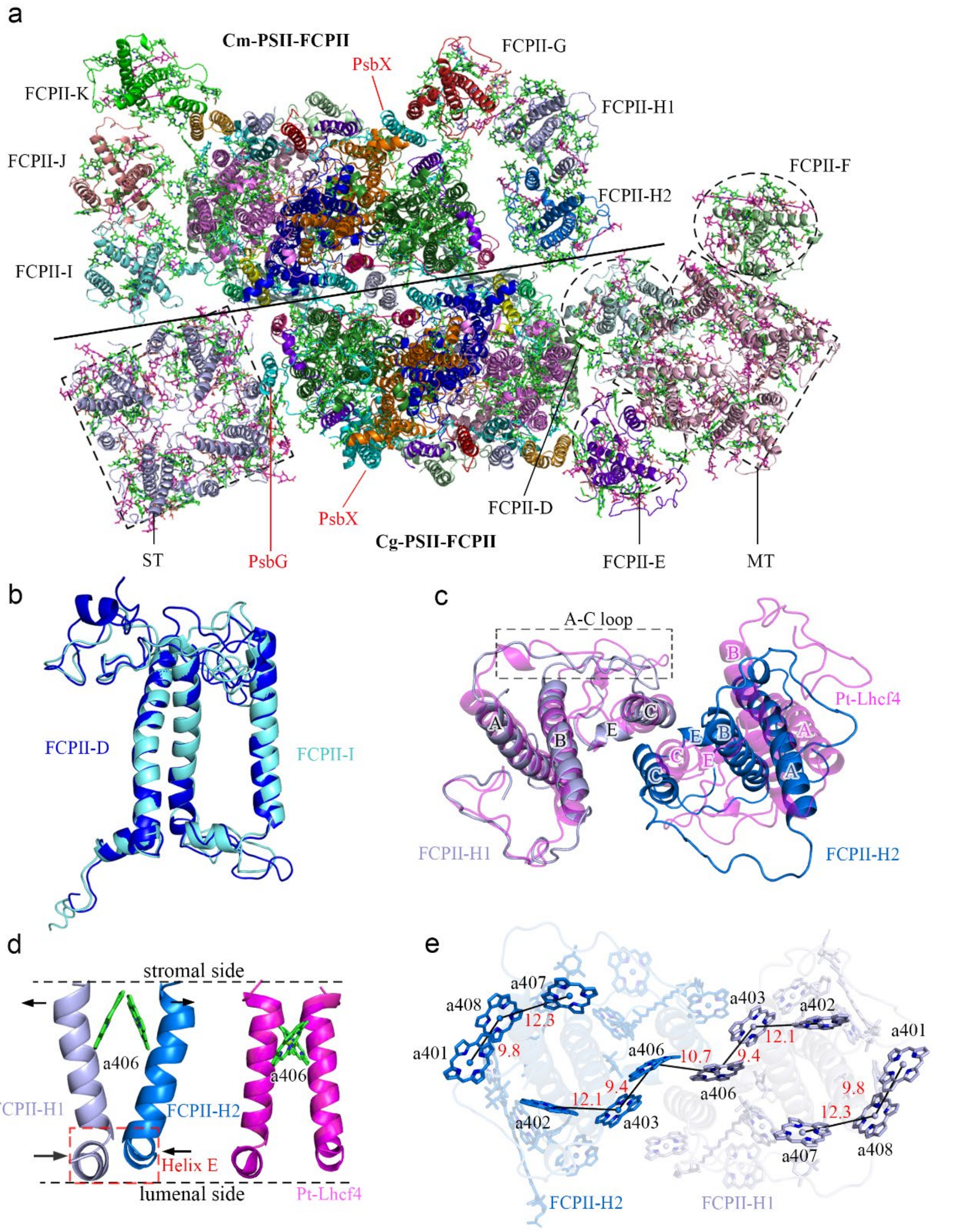

Supplementary Fig. 4. Comparison of the structure of Cm-PSII-FCPII monomer with those of Cg-PSII-FCPII monomer and Pt-Lhcf4. a, Overall comparison of structural models of a Cm-PSII-FCPII monomer (the top half) and Cg-PSII-FCPII monomer (the bottom half). PsbG present in the Cg-PSII-FCPII was not observed in the Cm-PSII-FCPII structure. **b**, Alignment of Cg-FCPII-D and Cm-FCPII-I. **c**, Overall comparison of two FCP dimers, Cm-FCPII-H1/H2 and Pt-Lhcf4. **d**, Comparison of coupled helices C in Cm-FCPII-H and Pt-Lhcf4. **e**, Distribution of stromal Chl clusters in FCPII-H.

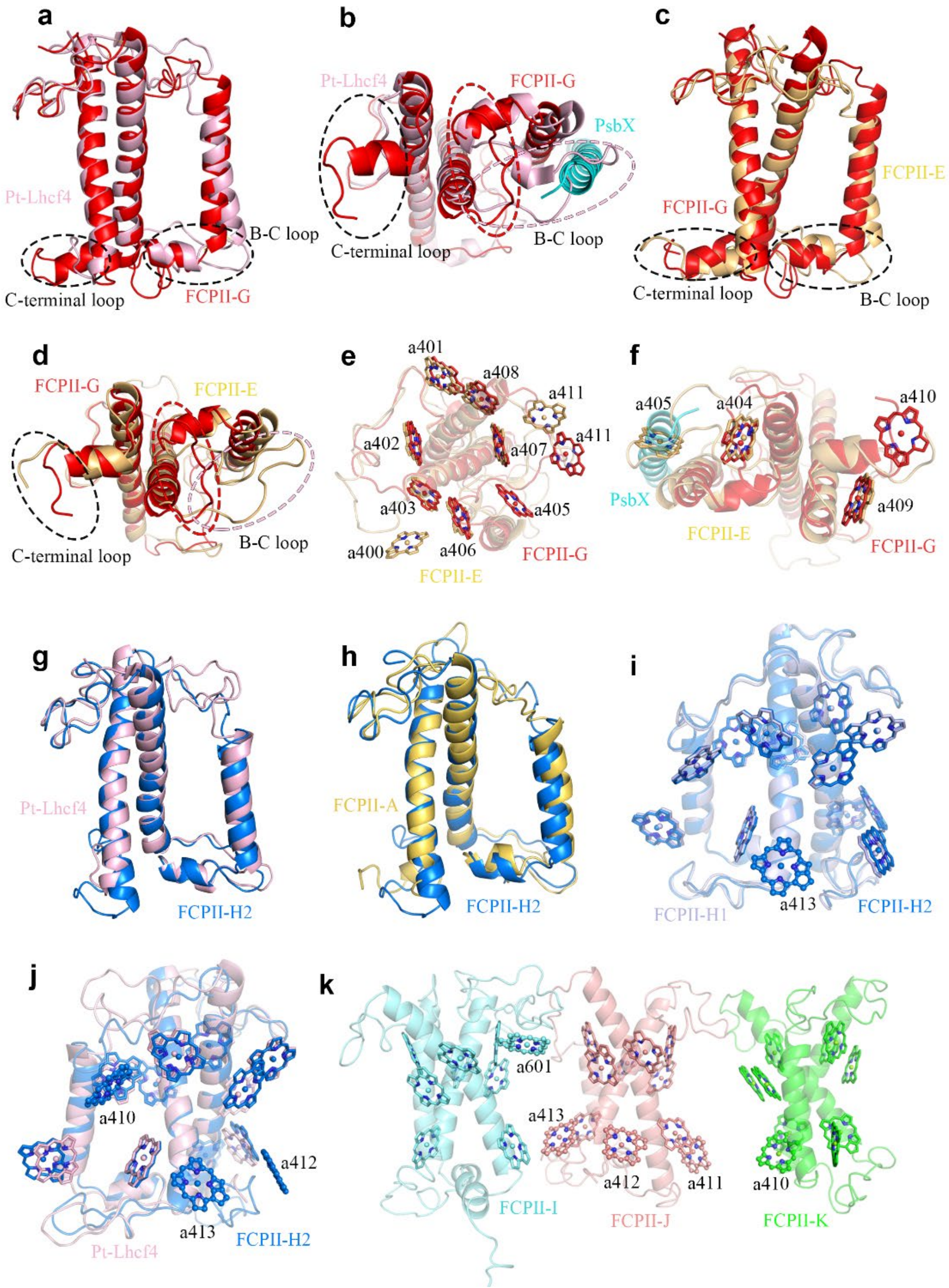

Supplementary Fig. 5. Comparisons of the structure of Cm-FCPs with those of Cg-FCPII-A and Pt-Lhcf4. a, **b**, Comparison of apoprotein structures between FCPII-G and Pt-Lhcf4. **c**, **d**, Comparison of apoprotein structures between Cm-FCPII-G and Cg-FCPII-E. Dashed ellipses highlight the different loop regions of Cm-FCPII-G. **e**, **f**, Comparison of Chls between Cm-FCPII-G and Cg-FCPII-E at the stromal and lumenal sides, respectively. Cm-PsbX forms intensive interactions with Cm-FCPII-G and makes Chl *a*405 shifted to the stromal side. **g**, **h,** Comparison of apoprotein structures between Cm-FCPII-H2, Pt-Lhcf4 and Cg-FCPII-A, respectively. **i**, Comparison of Chls between Cm-FCPII-H2 and Cm-FCPII-H1, showing the presence of an extra Chl *a*413 in Cm-FCPII-H2. **j**, Comparison of Chls between Cm-FCPII-H2 and Pt-Lhcf4. **k**, Presence of the extra Chls in FCPII-I/J/K in comparison with Pt-Lhcf4, which are important for lumenal energy transfer.

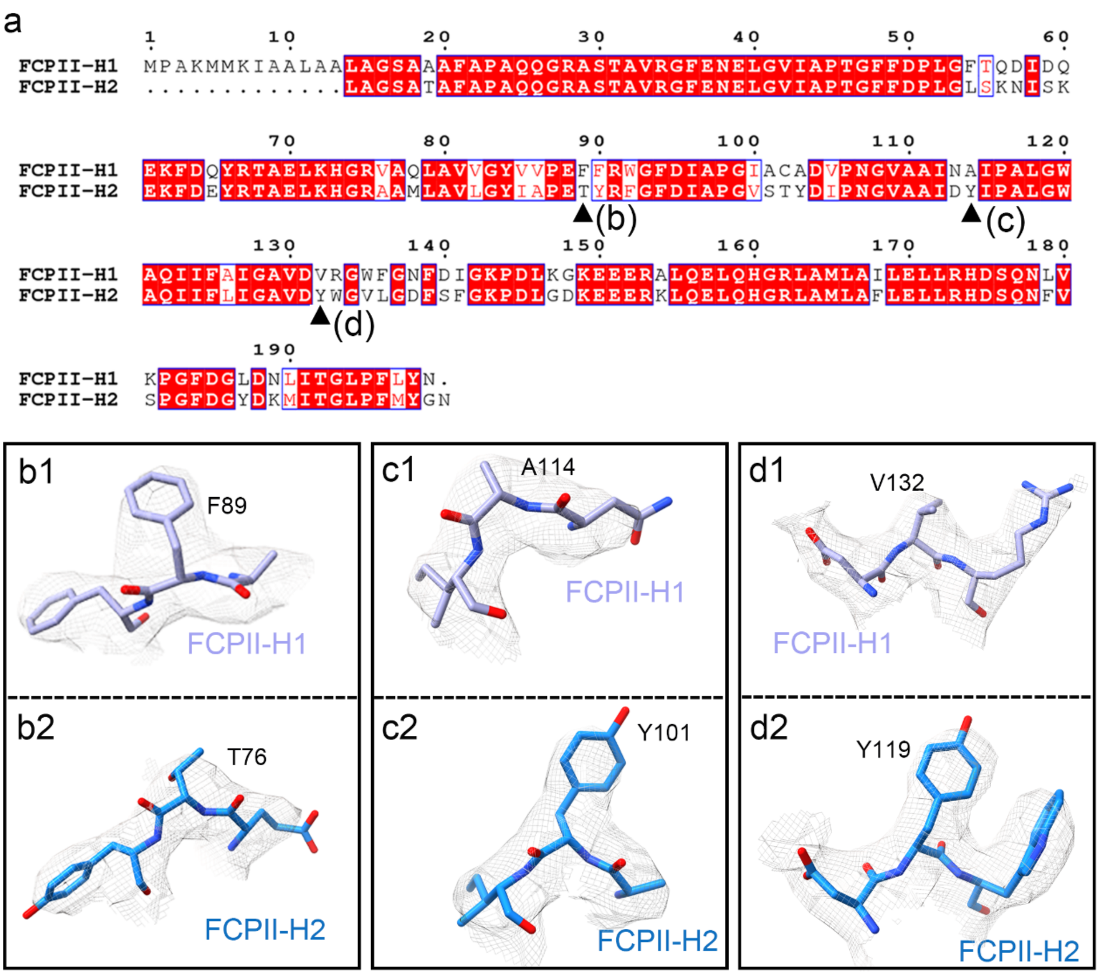

Supplementary Fig. 6. Sequence alignment of FCPII-H1/H2 and the assignment of the protein subunits into the structure of Cm-PSII-FCPII. a, Sequence alignment of Cm-FCPII-H1/H2. **b-d,** Assignment of FCPII-H1/H2 into the structure of Cm-PSII-FCPII by comparison of the map features of characteristic residues and the corresponding sequences.

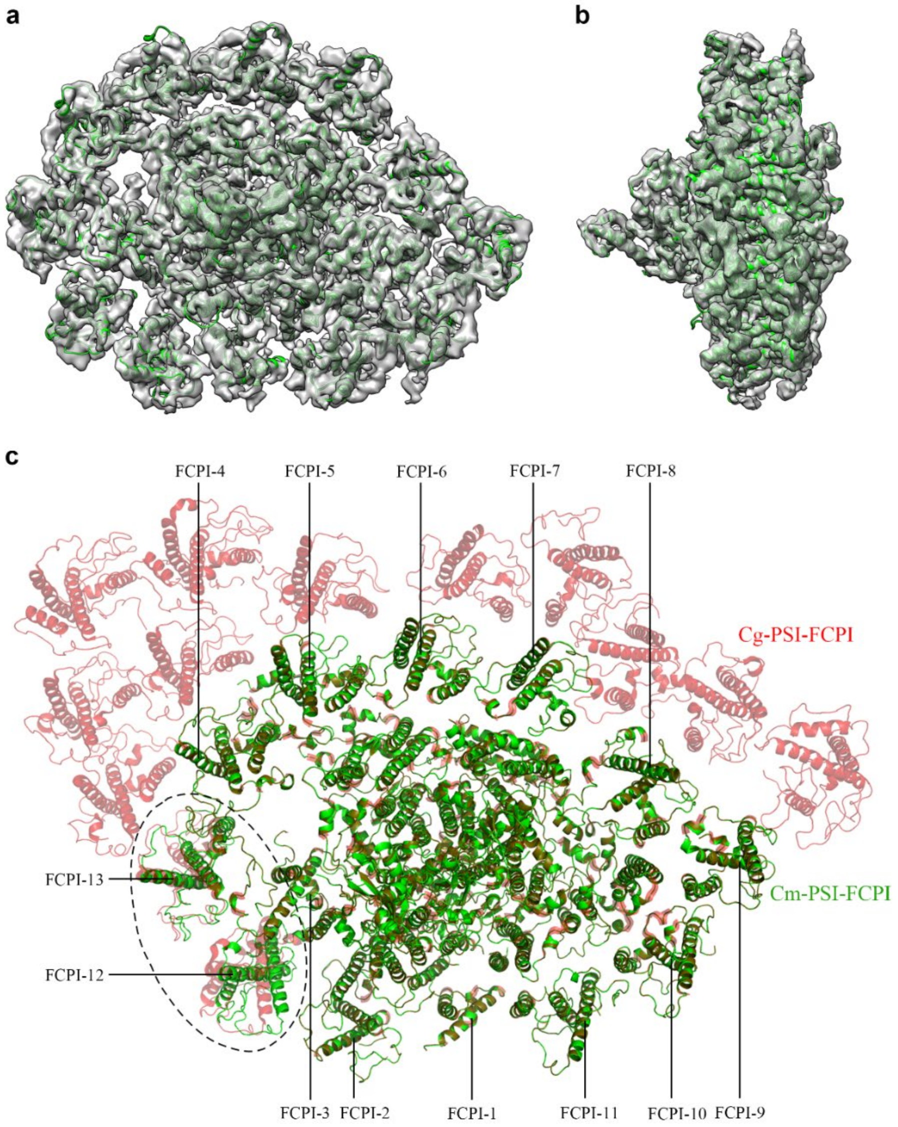

Supplementary Fig. 7. Overall structure of Cm-PSI-FCPI. a, **b**, Fit of structural models of Cm-PSI-FCPI with the density map viewed from the top **(a)** and side **(b)**, respectively. **c**, Comparison of structures between Cm-PSI-FCPI and Cg-PSI-FCPI, where a dashed circle indicates subunits where displacements occurred.

Supplementary Table 1. **Cryo-EM data collection, refinement, and validation statistics.**

Data collection and processing	PSII-FCPII	FCP trimer
Magnification	22,500 ×	81,000 ×
Voltage (kV)	300	300
Electron Exposure ($e^-/Å^2$)	50	60
Defocus range (μm)	-1.5 ~ -2.5	-1.0 ~-2.0
Pixel size (Å)	1.06	1.04
Symmetry imposed	C2	C3
Number of initial particles	1,192,896	1,471,158
Number of final particles	157,205	970,425
Map resolution (Å)	2.92	2.72
FSC threshold	0.143	0.143
Refinement		
Number of atoms	71446	7088
Protein residues	7682	543
Ligands	336	57
Root mean square deviations		
Bond lengths (Å)	0.006	0.006
Bond angles (°)	1.078	1.097
Validation		
MolProbity score	2.49	1.85
Clashscore	12.76	9.68
Ratamer outliers (%)	0.85	0.46
Ramachandran plot		
Favored (%)	90.82	95.16
Allowed (%)	8.34	4.84
Disallowed (%)	0.85	0.00

Supplementary Table 2. Pigments assigned to each subunit of Cm-PSII-FCPII and Cm-FCP05. * The pigment site 408 in FCPII-H and some sites in FCPII-I/J/K may potentially be assigned as Chls *c*. However, due to the limited resolution of the map, we temporarily assigned them as Chls *a*. Similarly, the Cars in FCP-I/J/K also need to be identified with higher resolution maps.

Subunits	Chl *a*	Chl *c*	BCR	Fx	Ddx	Dtx
D1	4		1			
CP47	16		3			
CP43	13		4			
D2	2		1			
PsbH			1			
PsbW	1					
PsbZ	1					
FCPII-G	11			2		1
FCPII-H1	12			2	2	
FCPII-H2	13			2	2	
FCPII-I	9			1	1	
FCPII-J	11			2	1	
FCPII-K	10			3		
**Cm-PSII-FCPII**	**206**	**0***	**20**	**24**	**12**	**2**
**Cm-FCP05**	**8**	**3**		**7**		

Supplementary Table 3. Correspondence of pigments numbering in the FCPII-G/H/I/J/K and FCP05 which described traditionally in this text and renumbered in the PDB data bank, respectively.

FCPII-G (Chain 0,5)		FCPII-H1 (Chain 1, 6)		FCPII-H2 (Chain 2, 7)		FCPII-I (Chain p, P)		FCPII-J (Chain 3, 8)		FCPII-K (Chain 4, 9)		FCP05 (Chain A)	
**Text No.**	**PDB No.**	**Text No.**	**PDB No.**	**Text No.**	**PDB No.**	**Text No.**	**PDB No.**	**Text No.**	**PDB No.**	**Text No.**	**PDB No.**	**Text No.**	**PDB No.**
301	301	301	317 (0, 5)	301	301	601	601	301	301	302	201	301	301
303	302	302	202	302	302	602	602	305	302	303	202	302	302
305	303	303	203	303	303	603	603	313	303	305	203	303	303
401	304	305	204	305	304	604	604	401	304	401	204	304	304
402	305	401	205	401	305	608	605	402	305	402	205	305	305
403	306	402	206	402	306	609	606	403	306	403	206	306	306
404	307	403	207	403	201 (1,6)	610	607	404	307	404	207	307	307
405	308	404	208	404	307	611	608	406	308	405	208	401	308
406	309	405	209	405	308	612	609	407	309	406	209	402	309
407	310	406	210	406	309	615	610	408	310	407	210	404	311
408	311	407	211	407	310	616	611	409	311	408	211	405	312
409	312	408	212	408	311			411	312	409	212	406	313
410	313	409	213	409	312			412	313	410	213	407	314
411	314	410	214	410	313			413	314			408	315
		411	215	411	314							409	316
		412	216	412	315							412	301(C)
				413	316							415	317
